# Visual acuity of the honey bee retina and the limits for feature detection

**DOI:** 10.1038/srep45972

**Published:** 2017-04-06

**Authors:** Elisa Rigosi, Steven D. Wiederman, David C. O’Carroll

**Affiliations:** 1 Department of Biology, Lund University, Sölvegatan 35, S-22362 Lund, Sweden; 2 Adelaide Medical School, The University of Adelaide, Adelaide SA 5005, Australia

## Abstract

Visual abilities of the honey bee have been studied for more than 100 years, recently revealing unexpectedly sophisticated cognitive skills rivalling those of vertebrates. However, the physiological limits of the honey bee eye have been largely unaddressed and only studied in an unnatural, dark state. Using a bright display and intracellular recordings, we here systematically investigated the angular sensitivity across the light adapted eye of honey bee foragers. Angular sensitivity is a measure of photoreceptor receptive field size and thus small values indicate higher visual acuity. Our recordings reveal a fronto-ventral acute zone in which angular sensitivity falls below 1.9°, some 30% smaller than previously reported. By measuring receptor noise and responses to moving dark objects, we also obtained direct measures of the smallest features detectable by the retina. In the frontal eye, single photoreceptors respond to objects as small as 0.6° × 0.6°, with >99% reliability. This indicates that honey bee foragers possess significantly better resolution than previously reported or estimated behaviourally, and commonly assumed in modelling of bee acuity.

The visual cognitive abilities of the honey bee, *Apis mellifera*, have few rivals among invertebrates. They can learn conceptual relations between stimuli, perform visual pattern categorization and even segregate the hierarchy of local versus global features within scenes^1^,^2^. Combined with the amenability of free-flying forager individuals for behavioural analysis, these abilities have contributed to bees becoming a major model for studying visual perception, learning and memory. Yet the physiological limits of achromatic vision in bees remain surprisingly unexplored^3^,^4^. Prior physiological studies of honey bee have only measured angular resolution from limited or poorly characterised regions of the retina, and in a dark adapted state^3^,^4^. These studies concluded that honey bees have a relatively modest average angular sensitivity of 2.6°. This value has been widely applied over the years when designing and interpreting behavioural experiments and ultimately for modelling flower perception^5^ and the effect of blur on the honey bee’s view of their world^6^,^7^.

Flower detectability and the smallest visual angle that a bee can perceive has also been investigated behaviourally, revealing that bees could not discriminate a dark object smaller than 3° when trained to fly to a sugar source in a double arm arena^6^,^7^,^8^. While these behavioural estimates for the lower limit to object detection seem to be reasonably well matched to prior physiological data, the fact that the latter were from eyes in a dark-adapted state might lead to underestimates of the true resolution^9^. For example, a widespread property of insect apposition eyes (including those of Hymenoptera) is the movement of pigment granules within the photoreceptors and/or pigment cells. These act together as a pupil that reduces the light flux upon light adaptation and also improves angular resolution^9^,^10^,^11^,^12^. We therefore recorded from photoreceptors adapted to bright light conditions, closer to those that foraging bees experience in nature (300 cd/m^2^). We estimated both the angular sensitivity Δρ (a measure of the receptive field size of single receptors, which limits resolution) and the smallest object a single cell can detect. Our results show that honey bee foragers have 30% better resolution than previous physiological estimates and a 5 times lower limit for feature detectability than has yet been estimated behaviourally.

## Results

### Angular sensitivity estimates

**Figure 1:**
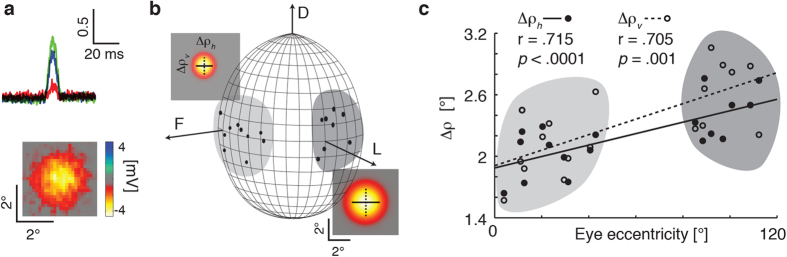
Intracellular measures of resolution in light adapted honey bee photoreceptors. (**a**) Example responses to red, green or blue bars swept through the receptive field (width: 4°, length 90°, velocity 80°/s) against a black background (responses are normalized to maximum response, i.e. to the white bar). All photoreceptors included in further analysis displayed similar spectral sensitivity with maximal response to green features. (i.e. green-sensitive or long-wavelength photoreceptor type). The raw 2-dimensional receptive field of a frontal photoreceptor was obtained by scanning its receptive field horizontally with a black square target (3 deg^2^) at a velocity of 32°/s across a vertical series of scan lines 0.13° apart. Colours show deflection in membrane potential in mV. (**b**) Estimates of individual photoreceptor receptive fields across the eye (*N* = 18). Ellipses denote relative sizes of angular sensitivity. Arrows denote frontal and lateral body axes (F: 0° azimuth, 0° elevation, L: 90° azimuth, 0° elevation) and the dorsal pole. The grey shading indicates lateral and frontal subsets of photoreceptors. Coloured plots show example 2D Gaussian kernel fits that account for the individual receptive fields from one frontal and one lateral photoreceptor, together with the vertical (dotted line) and horizontal (unbroken line) components of the angular half-width, Δρ. (**c**) Horizontal (⦁) and vertical (⚬) estimates of angular sensitivity (Δρ) in photoreceptors recorded across the eye as in (b). Least-square regression lines are reported for horizontal (unbroken line) and vertical (dotted line) components. Both Δρ_h_ and Δρ_v_ increase with increasing eccentricity, calculated as Euclidean distance from the frontal body axis (0° azimuth and 0° elevation).

To measure light adapted acuity, we recorded intracellularly from single photoreceptors while adapting the eye to a bright LCD monitor. We obtained data primarily for green sensitive cells, characterized by responses to coloured bars (green, red, blue) swept through the receptive field (Fig. 1a). We targeted cells within equatorial frontal and lateral sub-regions of the visual field likely involved in visual behaviours such as pattern discrimination and optical flow analysis^13^,^14^ (Fig. 1b). For each cell we estimated 2-dimensional receptive fields by recording responses to small, dark objects scanned raster fashion (left to right) across the receptive field. Resulting raw receptive field maps (Fig. 1a) served as input to a 2D Gaussian optical model that accounts for the size of the scanning feature^15^. This allowed estimation of Δρ, the full width at half maximum for horizontal and vertical components (Δρ_h_, Δρ_v_) of the underlying angular sensitivity (Fig. 1b).

Our Δρ estimates were generally symmetric about these two axes (Mann-Whitney U test, *U* = 303, *p* = 0.36, *N* = 18) with an overall average of 2.2° ± 0.3° for Δρ_h_ and 2.3° ± 0.4° for Δρ_h_. Consistent with optical measurements suggesting a frontal eye region with higher acuity^16^, we found that Δρ_h_ and Δρ_v_ both increase away from the front of the eye (Fig. 1c). Our data suggests an average frontal Δρ of 1.9°, estimated from the intercept between the linear regression and the frontal mid-line (Fig. 1c). Individual cells just ventral to the frontal body axis had Δρ as small as 1.6°. Our data clearly indicates at least 30% higher resolution than prior estimates from honey bee retina^3^,^4^.

### Feature detectability threshold

**Figure 2:**
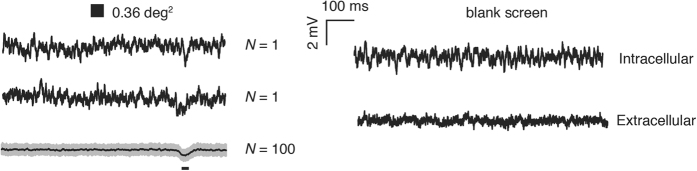
Measures of photoreceptor noise for estimation of the feature detectability threshold. (**Left**) Single and averaged (±σ) raw response of a frontal photoreceptor (azimuth, 27°; elevation, −16°) when a near-threshold dark object (0.6° × 0.6°) was swept across its receptive field (*v* = 65°/s). The black bar below the averaged responses denotes the approximate receptive field of the cell (defined here by its acceptance angle, Δρ) transformed into the time domain. **(Right)** In order to quantify the contribution of extracellular/instrument noise estimates, noise threshold was calculated as standard deviation of the intracellular responses to a blank screen corrected for the responses to the same stimulus obtained right outside the cell.

**Figure 3:**
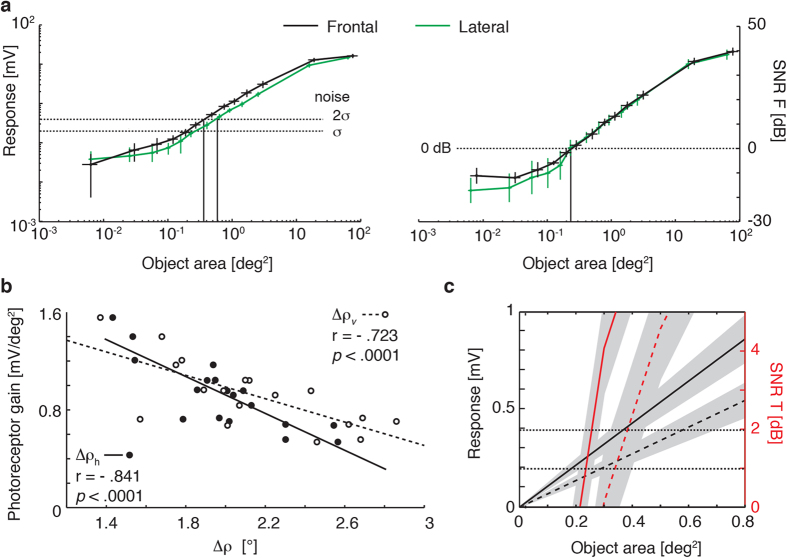
Measures of object detectability thresholds for single photoreceptors. (**a**) **Left** Peak responses (in mV, mean ± 95% confidential intervals, CI) for increasing object areas in both frontal (*N* = 10, black line) and lateral photoreceptors (*N* = 8, green line). Horizontal dashed lines represent σ and 2σ for the averaged photoreceptor noise (see experimental procedures and Fig. 2). **Right** Mean ± 95% CI SNR estimated in the frequency domain for frontal (black line) and lateral (green lines) photoreceptors. Horizontal dotted line represents 0 dB. Vertical lines are drawn to identify the corresponding object size at 2σ noise and for an SNR of 0 dB respectively. (**b**) Photoreceptor gain obtained as slope from the linear regression of responses to objects <1° (as in the right panel) correlate with both the horizontal and vertical measures of Δρ. (**c**) Zoomed in view of the data in (a), for object areas ≤0.8 deg^2^. Black lines are the linear regression for responses to objects <1° while data in red are for the signal to noise ratio (SNR) calculated as described in ‘Temporal analysis of SNR’. Dotted and unbroken lines represent average lateral and frontal photoreceptor responses, respectively. Horizontal dotted lines represent σ and 2σ of the averaged photoreceptor noise. Means ± 95% CI are indicated by shading.

Our angular sensitivity estimates provide direct quantification of the optical blur in sampling of scenes by photoreceptors. But how this translates into potential detectability of features also depends on receptor noise limits. We therefore recorded deflections in membrane potential induced by different sized black, square objects (from 0.01 deg^2^ to 64 deg^2^) drifted through the receptive field centre and compared this with the underlying receptor noise (recorded from the same cells when viewing the blank, white screen, Fig. 2). In both frontal and lateral regions, responses saturate for large objects that fill the receptive field but decrease linearly as the object area falls below 1 deg^2^ (Fig. 3a). As features fall below the size of the receptive field they are increasingly blurred to a lower effective contrast until the response is indistinguishable from noise^15^, so linear fits to this threshold region provide a measure of receptor gain and contrast sensitivity. We found the slope of such fits to depend strongly on Δρ, (smaller Δρ = steeper slope, Fig. 3b) suggesting that it primarily reflects acuity rather than regional differences in transduction gain. The intersection of this function with the noise of the photoreceptor determines the lower threshold for a detectable object. At a threshold of 2x the standard deviation (σ) of the noise (Fig. 3a and c), our data predict a minimum detectable target size of just 0.35 deg^2^ frontally (i.e. an object subtending 0.59° × 0.59°) and 0.55 deg^2^ laterally. In confirmation of these threshold measures, we also estimated the signal-to-noise ratio (SNR) as a function of target area from the average power spectral density of single responses versus noise spectra (Fig. 3a) as well as SNR from average peak time responses over noise (Fig. 3c). These reveal a useful SNR (>0 dB equivalent to 1:1 signal:noise) for targets below 0.3 deg^2^ both frontally and laterally. Thus, we found that even single photoreceptors have sufficient acuity and contrast sensitivity to detect objects around 0.5° across - substantially smaller than the angle subtended by each ommatidium. This estimate is at least 5 times smaller than the smallest features demonstrated behaviourally^6^,^7^,^8^.

## Discussion

Although the visual acuity in honey bees has been investigated since the first decades of last century^17^, our data suggest it has been underestimated in prior work, both in terms of the angular sensitivity function and the minimum feature sizes resolvable by the retina. One possible factor reconciling the smaller estimates of Δρ (i.e. higher acuity) that we found compared with prior physiological studies might lie in differences in the photochemical and mechanical dynamics underlying the different adapted states of the eye, i.e. from dark-adapted (as used in previous physiological studies) to light-adapted (as in the present study)^9^,^18^. In other insects, for example, estimates of Δρ have been shown to vary with the light adaptation level by almost a factor of 2 in some cases^9^. This may reflect a widespread property of light adaptation in insect apposition eyes (including those of Hymenoptera) where either primary pigment cells constrict the effective diameter of the proximal crystalline cone, or retinula cell pigment granules migrate towards the rhabdom tip^11^,^12^,^19^. In both cases, the resultant constricted pupil leads to improved angular resolution^11^,^12^,^19^.

Another possible factor in the higher acuity we observe compared with prior physiological studies was our success in obtaining multiple recordings from photoreceptors in the frontal-ventral acute zone (Fig. 1c). This region, where we observed smaller Δρ values, is one also associated with a higher sampling density of ommatidia typical of flying insects^20^. In honey bees, evidence for such an acute zone comes from optical estimates of smaller inter-ommatidial angles that sample this part of the scene^16^,^21^, correspondingly larger ommatidial facets^22^, and through behavioural evidence for regional differences in visual performance^13^. The optical data suggests that bees have inter-ommatidial angles (Δϕ) of 2.1° (horizontally) and 0.9° (vertically) in this eye region^21^, although it has been argued that geometric distortions of the underlying photoreceptor mosaic lead to an average around 1.7° in both dimensions^16^, a value more consistent with behavioural estimates for grating resolution^23^. Our data (with Δρ in this region below 1.9°) are thus a better match for the optical sampling resolution of the honey bee acute zone^16^,^21^ than prior physiological estimates. The closer match between Δρ and Δϕ suggested by our findings are indicative of mild optical undersampling, as is more typical of diurnal insects with apposition eyes than the oversampling suggested by the older estimates of Δρ^20^. One advantage of such mild undersampling in an apposition compound eye is better contrast transfer between the fine details of a visual scene and its retinal image^24^,^25^. This might be advantageous during flying manoeuvres by increasing the contrast gain for fast moving features^20^,^24^. Improved resolution and contrast transfer also improves the degree to which very small objects might still be detectable in the resulting averaged intensity across the receptive field, even once they are smaller than the optical sampling and thus not ‘resolved’ *per se*^15^.

One behavioural demonstration of such sub-pixel ‘hyperacuity’ comes from the honey bee drone, which Vallett and Coles^26^ showed could react to a black ‘queen dummy’ object as small as 0.41° - considerably smaller than their estimates of the photoreceptor angular sensitivity. They estimated that at this behavioural threshold, the effective contrast of the target was just 8%, even though it was a black object seen against a bright background^26^. This finding underscores the fact that the resolution limit of the eye is set not just by the quality of the optics, but also by the noise levels of the detectors. Objects smaller than the angular sensitivity of the photoreceptors become effective ‘point source’ objects that form a similar image on the retina except for their reduced apparent contrast, which continues to decline with decreasing object size. Hence ultimately this task is limited by the signal to noise ratio^15^,^27^.

Our physiological estimate of a lower detection threshold for small features in forager honey bees of 0.6° is almost 50% larger than the behavioural estimate for their male counterparts, which have larger eyes and optics specialised for queen pursuit^22^,^28^. Nevertheless, it is still an impressive value, well below the average frontal angular sensitivity half-width of 1.9°. Prior behavioural analysis of free-flying forager female bees conducted in a two-choice maze found that the smallest achromatic dot that bee can be trained to recognize (when associated to a sugar reward) is 3° at an object contrast of 87% 8. Subsequent behavioural studies in a Y maze estimated a detectability threshold for achromatic targets of 3.7°–5° 6,7. These studies thus indicate a greater than 5-fold discrepancy compared with our physiological estimate of minimum resolvable target size. This discrepancy is all the more surprising considering that our data are for single photoreceptors: a substantial further improvement in signal gain (or noise reduction) would be expected from downstream summation of lamina neurons that feed into behaviour, which each receive synaptic input from at least 8 photoreceptors in bees^29^.

The much better match between drone bee behaviour and optics might result in part from the intrinsic motivation of drone bees for performing a biologically relevant task (i.e. tracking a small moving object). The minimal resolvable angles obtained so far in behavioural studies of foragers might thus be limited more by the behavioural paradigm and lack of motivation in the animal, than by optical or physiological constraints. Typical natural scenes in which workers naturally forage would contain a rich array of larger features, so single small targets close to the limits of resolution would not be intrinsically salient in the way that such stimuli are for drones. Also, as we noted earlier, an object subtending angles below the angular size of an ommatidium would be rendered to a lower effective contrast in the retinal image than a larger object^15^,^27^ and could thus present a different cue in terms of its (apparent) contrast to that of a larger object.

Our finding of higher visual acuity than suggested by prior studies of a well-studied species should prompt reconsideration of single photoreceptor visual acuity among a range of other diurnal insect species. It also suggests a need for revision of models used in predictions of what bees can resolve in a given scene for biologically relevant tasks such as identification of food sources, particularly given recent studies highlighting the relevance of feature detectability in navigation^30^.

## Methods

### Animals

Honey bee foragers (*Apis mellifera, N* = 14) were collected in the Adelaide Botanic Garden (SA, Australia) from November 2014 to May 2015.

### Electrophysiological recordings

After immobilizing at 4 °C for few minutes, bees were inserted into an Eppendorf tube cut at its narrow end, and mounted on a metal stage. Head, mouthparts and antennae were waxed with a 1:1 mixture of rosin and beeswax to avoid movements during recordings. A triangular shaped window on the dorsal area of the left cornea was cut in order to access the retina. The open cut was immediately sealed with vacuum grease to avoid desiccation. Intracellular membrane potentials of single photoreceptors were recorded using an amplifier with a low noise, high-input impedance headstage (NPI BA-03X). Sharp electrodes were fabricated from aluminosilicate glass capillaries (SM100F-10, Harvard Apparatus) pulled in a Sutter Instruments P-97 puller and filled with 2 M KCl solution. Electrode resistance was 80–240 MΩ. A piezo controlled manipulator allowed stepping through the retina with a 5–7 μm step size. Successful penetration of photoreceptors was indicated on the basis of the membrane potential responses to full screen flicker stimuli. As a criterion for healthy recording, photoreceptors that showed less than 12 mV responses to dark full screen flicker were discarded from the analysis. 18 photoreceptors were used in the analysis.

### Visual stimuli

A liquid crystal display monitor (1920 × 1080 pixels, EIZO Foris FG2421) was placed 150–235 mm in front of the animal. The eye was light adapted to the white full screen display (300 cd/m^2^) as a default experimental background, except during brief characterization of spectral sensitivity (see below).

#### Photoreceptor characterization

In order to characterize single photoreceptors, the response to a full screen flicker stimulus was recorded for 20 s (Temporal frequency 1 Hz) at least once for each cell. The likely spectral class of the cell was estimated by presenting a moving coloured vertical bar (4° × 90°, left to right, velocity: 80°/s) against a black background, with blue (B), green (G), red (R) and white (R + G + B) bars presented in randomized order.

#### Receptive field (RF) scans and angular acceptance estimation (Δρ)

The centre location of the receptive field was initially estimated from responses to black bars (4° × 90°, velocity: 80°/s) moved across the screen along the 4 cardinal directions. Online analysis of these responses then allowed automatic generation of a stimulus sequence to scan a small region of interest (ROI, 17° square) centred on the receptive field. During subsequent scans a small black square (1.7° × 1.7°, Weber contrast = −0.998) drifted left to right (velocity 40°/s) within ROI at 101 sequential raster lines (i.e. vertical scanning resolution 0.17°). With a pre stimulus recording time of 0.5 s the total scanning time required to obtain an RF was approximately 120 s. The data obtained fed a model that allowed us to fit the measured receptive fields by convolving a 2D Gaussian kernel with the stimulus target^15^. Horizontal and vertical Δρ values were then estimated from the best-fit kernel. The same model also allowed us to identify the exact centre coordinates of the RF for subsequent experiments. In fitting a 2D Gaussian (linear) kernel we assume linearity in the measured signal. Despite the action of voltage-gated conductances, a linearized model has recently been shown to accurately describe insect photoreceptor responses below 7mV^31^. We therefore selected a target size for the black object small enough to limit maximum responses below 5.5 mV. We further confirmed the linear range in the honey bee by measuring photoreceptor responses to a larger target (21° × 21°) over a range of grayscales (logarithmically spaced RGB values). Responses below 6 mV are well-fitted by linear regression (r = −0.991 ± 0.007; *N* = 2).

#### Contrast sensitivity

Black square objects varying in size were drifted left to right through the centre of the RF at a velocity of 80°/s. The target linear dimensions were varied by a logarithmically spaced series from 0.1° to 8.0° (corresponding to target areas from 0.01 deg^2^ to 64 deg^2^, Weber contrast = −0.998) in random order. Each target was presented 50 to 150 times in total for each cell. The RF scan (see above) was calculated before and after each sequence in order to confirm that targets were centred on the RF.

### Analysis

All analysis used Matlab 2014b. For each bee the visual angle subtended by each pixel (α) was calculated as follows:


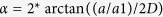


where *a* is the screen width in centimetres, *a1* the screen width in number of pixels, and *D* is the distance from the screen in centimetres.

#### Receptive field location

For each cell we calculated the coordinates of the receptive field location on the eye as distances from the central point (0° azimuth, 0° elevation), namely the midpoint in front of the eyes and perpendicular to the dorsal head axis. These distances were first measured in pixels and then transformed in degrees using the degree/pixel ratio calculated for that cell. Eccentricity was calculated as the Euclidian distance between the centre (0°, 0°) and the coordinates in degrees of the RF centre.

#### Contrast sensitivity

Average unstimulated membrane potential was subtracted before filtering (in both forward and reverse directions to negate any phase shift) with a 3^rd^ order lowpass butterworth filter (cutoff 70 Hz) and averaging responses to 50–100 stimuli. For each target size, responses were estimated as the maximum membrane potential (i.e. peak hyperpolarization) in the filtered, averaged response during a small window centred on the target passage across the receptive field (confirmed in trials where a response was clearly evident above noise). To compare the different contrast sensitivity curves we calculated the slope of a linear regression model fitted to the response for object areas smaller than 1° (Fig. 3c).

#### Noise estimates

To estimate noise levels in each photoreceptor, the membrane potential to a full white screen was recorded for 20 seconds. An additional 20 s full white screen response was also recorded extracellularly immediately after stepping out of the cell after the entire experiment was completed (*N* = 15). This allowed estimation of the contribution of amplifier/instrument noise to recorded noise estimates. Signals were band-pass filtered (3^rd^ order butterworth filter, passband 0.5–200 Hz). Final noise levels were estimated as the difference between the averaged standard deviation of the intracellular and extracellular responses to the same blank stimulus^32^.

##### Frequency analysis of SNR

Intracellular and extracellular responses to the white screen were bandpass filtered (3^rd^ order butterworth filter, passband 0.5–100 Hz) with the DC offset subtracted. The average noise power spectral density in the 5–15 Hz frequency range was then estimated using the bandpower.m function in Matlab.

A similar analysis estimated signal power in the same frequency range after first averaging away noise from the signal component of the response to identical trials.

##### Temporal analysis of SNR

An additional SNR was calculated as comparison (data in red, Fig. 3c); for each target area the SNR was obtained as 10*log10(signal^2^/noise^2^), where signal was the peak hyperpolarization and the noise was the cell specific noise as obtained as described above. This allowed us to estimate SNR (±95% confidential intervals) in single cells rather than obtaining a mean value for the averaged noise level across cells.

##### Statistical analysis

As the Δρ values did not meet the assumptions of normality, we used a two-tailed Mann-Whitney U test (Matlab, 2014b) to compare horizontal and vertical components of Δρ.

Both stimuli and analysis were made by custom built scripts in Matlab 2014b.

## Additional Information

**How to cite this article:** Rigosi, E. *et al*. Visual acuity of the honey bee retina and the limits for feature detection. *Sci. Rep.* **7**, 45972; doi: 10.1038/srep45972 (2017).

**Publisher's note:** Springer Nature remains neutral with regard to jurisdictional claims in published maps and institutional affiliations.
